# Chronic *E. Coli* Drug-Resistant Cystitis Treated with a Sequence of Modulated Extremely Low-Frequency Electromagnetic Fields: A Randomized Study of 148 Cases

**DOI:** 10.3390/jcm13092639

**Published:** 2024-04-30

**Authors:** Ivan D’Agostino, F. Marelli

**Affiliations:** 1 Independent Researcher, ID Medicals, 00163 Rome, Italy; 2 Independent Researcher, CRESO LLCs, 6830 Chiasso, Switzerland

**Keywords:** EMF (electromagnetic field), ELF (extremely low frequency), PEMF (pulsed electromagnetic field), *E. coli* (*Escherichia coli*), BSBI (signal sequence), osteopathic palpation, osteopathic assessment, osteopathic landmarks

## Abstract

**(1) Background:** This study investigated the effects of sequenced electromagnetic fields, modulated at extremely low frequencies and intensities, in the treatment of drug-resistant *Escherichia coli* (*E. coli*)-induced chronic bacterial cystitis. **(2) Methods:** A total of 148 female participants, aged 18 to 80 years diagnosed with chronic bacterial cystitis caused by drug-resistant *E. coli*, were recruited for this study. Participants were randomly assigned to two groups: an experimental group (n = 74) with osteopathic palpation and assessment treated with a sequence of electromagnetic fields, and a control group (n = 74) receiving a placebo treatment. Both groups were assessed at this study’s outset, 4 weeks after eight applications, and at 12 weeks for symptomatic presentation and laboratory parameters. **(3) Results:** After 4 weeks of treatment, a significant difference was observed between the two groups regarding D-DIMER levels, IL-6 levels, erythrocyte levels, leukocyte levels, and *E. coli* levels (*p* < 0.001). By the 12th week, the experimental group continued to exhibit a significant reduction in the examined parameters compared to the control group (*p* < 0.001). Additionally, the treatment did not induce any side effects in the patients in the experimental group. **(4) Conclusions:** Treatment with coherently sequenced electromagnetic fields, modulated at an extremely low frequency and intensity, not only appears to provide an effective alternative for the symptoms of chronic bacterial cystitis caused by drug-resistant *E. coli* but also demonstrates a potent antibacterial effect.

## 1. Introduction

Cystitis refers to the infection of the lower urinary tract, more specifically, the urinary bladder [1,2]. It can be broadly classified into two main types: uncomplicated and complicated. Uncomplicated cystitis pertains to lower urinary tract infection (UTI) in otherwise healthy individuals. On the other hand, complicated cystitis is associated with risk factors that increase the likelihood and severity of infection or the potential for antibiotic therapy failure. Acute cystitis is typically caused by a bacterial infection of the urinary bladder. Women are particularly susceptible due to the proximity of the rectum to the urethral meatus, as well as the relatively short length of the female urethra. *Escherichia coli* (*E. coli*) is the most common etiological agent in uncomplicated UTIs in women, accounting for approximately 75% to 95% of cases, followed by *Klebsiella* [3].

Similarly, *E. coli* is also the most common cause of complicated cystitis. However, the spectrum of microbial pathogens that can cause complicated urinary tract infections (UTIs) is much broader and includes organisms such as *Enterobacter*, *Citrobacter*, *Serratia*, *Pseudomonas*, *enterococci*, *staphylococci*, and even fungi. The incidence of antimicrobial resistance in complicated infections is also significantly higher. Notably, resistant organisms include broad-spectrum beta-lactamase-producing bacteria (ESBL), as well as organisms resistant to carbapenems and fluoroquinolones, particularly *E. coli* [4]. Chronic inflammation, triggered by increasing levels of oxidative stress, is implicated in the development of bacterial infections, leading to tissue fibrosis. Moreover, *E. coli* appears to be resilient to oxidative damage due to the corrective effect of alanyl-tRNA synthetase (AlaRS), which remains unaffected by oxidative stress, thus protecting the bacterium [5,6,7,8,9,10].

Chronic inflammation of the urinary tract can result in fibrosis development. Fibrosis is caused by the upregulation of collagen I and III genes, fibronectin, and TGFβ1, the downregulation of WNT11, and the production of YKL-40 by mast cells in interstitial cystitis. Based on these similarities, it can be hypothesized that YKL40 may also play a key role, primarily due to the abundance of mast cells present in all forms of cystitis. Fibrosis invades the detrusor and lamina propria, particularly around blood vessels, making them stiffer. The consequence is a decrease in bladder functionality and difficulties in contractions, leading to spasms, pain, and voiding difficulties. This mechanism could also influence the drug resistance of *E. coli*, given its facultative anaerobic nature [11]; thus, fibrosis (in which the extracellular matrix, ECM, is also involved) could decrease hypoxia tolerance, promoting *E. coli* resistance and shielding it from the effects of antibiotics.

The growing issue of antibiotic resistance has heightened the need to explore different therapeutic methods, prompting a reconsideration of pathology in relation to tissue mechanics, electrical gradients, and tensional changes [12,13,14]. One emerging area is biophysics, particularly the exploration of sequenced electromagnetic fields (EMFs) modulated at extremely low frequencies (ELFs). As modern technology advances, the question of how EMFs influence biological processes becomes increasingly relevant.

Numerous studies have investigated these interactions on various scales, revealing that an ELF has anti-inflammatory and anti-edematous effects. These effects are achieved through the modulation of cytokines, the impact on vascular walls, the remodeling of the extracellular matrix, increased gene expression of Heat Shock Proteins (HSPs) [15], and the repolarization of cellular membranes. This is facilitated by a sequence of magneto-electric codes capable of collectively counteracting the reactive effects of the tissue [16,17]. ELF systems have been found to decrease levels of pro-inflammatory cytokines, such as TNFα, IL-1β, IL-6, and IL-8 [18,19,20], modulate pro-inflammatory cytokines [21,22], restore membrane Ca-ATPase function [23], facilitate electroconformational coupling of transmembrane α-helices (aquaporins) [24,25], increase local circulation [26], and alter the redox state of the extracellular matrix by displacing membranous electrostatic charges [27,28].

The effect on edema is supported by the forced vibration of transmembrane proteins due to the transfer of charges and phonons. The electroconformational coupling of alpha helices and beta barrels of aquaporins can modify both proton flow and forced water flow, substantially altering the pH dilution of intra- and extracellular solutes and the redox state of the extracellular matrix. This same mechanism of action also modulates ionotropic and metabotropic receptors of the cell, thereby influencing short- and long-distance cellular signaling [24,25]. There is evidence of an antioxidant effect as well [29,30].

Recent research suggests that EMFs may have antibacterial effects, providing a potential mechanism of action against resistant bacteria [31,32]. While EMFs have been investigated in various medical contexts, their application in managing cystitis is a relatively unexplored area. Therefore, this study aimed to investigate the effectiveness and antibacterial, immunomodulatory, and anti-inflammatory effects of ELFs according to the osteopathic fundamentals of palpation and assessment of the treatment of drug-resistant *E. Coli*-induced chronic bacterial cystitis.

## 2. Materials and Methods

### 2.1. Subjects

Patients (women, aged 18 to 80 years, mean age 38 years, MED = 38.5) suffering from chronic cystitis (at least 3 episodes of cystitis per year) and infected with drug-resistant *E. coli* were included in this study. Participants were randomly assigned to either the experimental group or the control group in a 1:1 ratio. The included participants had previously taken trimethoprim, nitrofurantoin, fluoroquinolones, sulfonamides, amoxicillin, and ampicillin, as well as dietary supplements, like d-mannose and cranberry, without satisfactory clinical response. The patients analyzed suffered from recurrent chronic cystitis, including burning during urination, after sexual intercourse, after dietary changes, and in stressful situations.

### 2.2. Methods

All participants underwent testing for IL-6 and D-dimer (from a complete blood count), urine analysis (pH, specific gravity, urobilinogen, red blood cells, and leukocytes), and urine culture. Urine analysis was conducted using refractometric and cytofluorimetric methods following SIBioC guidelines, and urine culture was performed using a conventional method according to AMCLI and EUCAST guidelines. Participants were assessed at the baseline, at the end of the intervention (4 weeks), and at 12 weeks after the intervention. The within-subjects factor was time, with three levels: Time 1, Time 2, and Time 3. The between-subjects factor was group, with two levels: control and experimental.

### 2.3. Treatment

For treatment, a sequence of signals (BSBI) was created, combining frequency, field intensity, waveform, application time, and pulsation time. The created BSBI sequence underwent artificial intelligence processing (ChatGPT 3, https://openai.com accessed on 2 January 2023) to find a regular rhythm, avoiding abrupt changes that could impede signal clarity.

**Figure 1:**
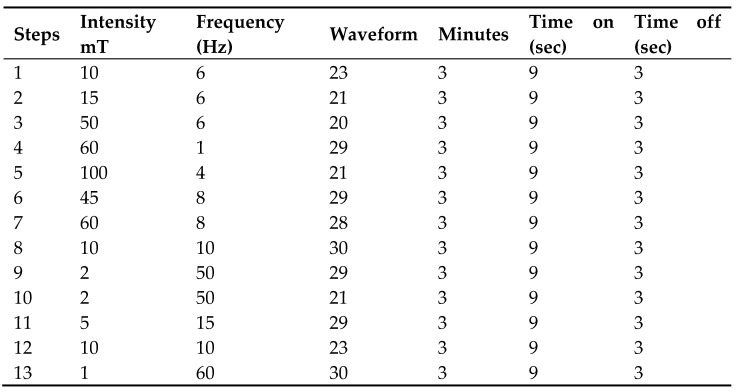
BSBI sequence.

In order to emit the BSBI sequence, a PEMF ion resonance generator, Seqex (SISTEMI Srl) mod FAM (compliant with Directive 93/42/EEC and standard EN 60601-1 regarding safety and essential performance, CE Certificate issued by CE0051), was used. The applicators LIE (M.F.I. Srl), consisting of two emitters, were used. The PEMF ion resonance generator can write signal sequences (combining frequency, field intensity, waveform, application time, and time per cycle), store them on a card, and emit them. Emitted sequences were from 1 to 80 Hz, with 30 waveforms, and signal intensity was from 1 to 100 µT at the source. Each LIE emitter weighs 125g and has a conical range starting from 5–6 cm up to 15 cm, with a depth of about 20 cm (Figure 1).

A palpation assessment (local visceral listening) of the bladder (bladder fascia, bladder ligaments, umbilical ligaments, urachus), rectum, and sigmoid colon was performed. Osteopathic palpation revealed that the evaluated tissues were all very rigid, with a marked restriction of mobility of the Douglas pouch in all participants assessed.

The BSBI sequence consisted of 13 steps, each step involving a different combination of frequency, intensity, and waveform. The action time was 3 min for each step, with a total duration of 39 min for the entire sequence. The entire sequence was applied to the experimental group, while a placebo was used on the control group by placing the applicators without emitting signals.

**Figure 2:**
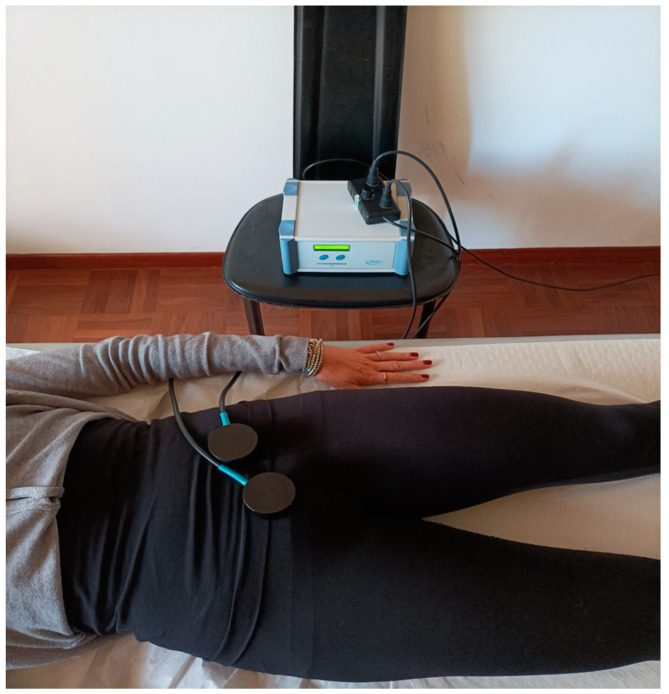
Treatment with the BSBI sequence.

A total of 8 applications were performed twice a week, with a 48–72 h interval between each session (e.g., Monday and Thursday) (Figure 2). The patients did not take antibiotics or other medications during the treatment.

### 2.4. Statistical Analysis

A two-way repeated measures ANOVA design was used to investigate the effects of time and group on the dependent variables. Descriptive statistics (mean, standard deviation, minimum, and maximum) were calculated for BMI, pH, specific gravity, and urobilinogen. Five two-way repeated measures ANOVAs were performed to test the effects of group (control vs. experimental) and time (Time 1 vs. Time 2 vs. Time 3) on each dependent variable (D-DIMER, IL-6, erythrocytes, leukocytes, and *E. coli*). The Greenhouse–Geisser correction was applied when the assumption of sphericity was violated. The effect sizes were reported using partial eta-squared values.

## 3. Results

### 3.1. Demographics

A total of 146 participants were eligible for this study. Participants were divided in a 1:1 ratio in both the experimental (n = 74) and control group (n = 74). The mean BMI was 21.26 (SD = 1.66), with a range of 18.5 to 24.2. The mean pH was 6.64 (SD = 0.70), with a range of 5.5 to 7.9. The mean specific gravity was 1015.58 (SD = 5.63), with a range of 1006 to 1025. The mean urobilinogen mg/dL was 0.81 (SD = 0.45), with a range of 0 to 1.5 (Table 1).

### 3.2. D-DIMER Levels (ng/mL)

There was a significant main effect of time, F (1.86, 272.01) = 1194.682, *p* < 0.001, η2 = 0.891, indicating that the mean D-DIMER level changed significantly across the three time points. The within-subject contrast showed that this effect was quadratic, F (1, 146) = 352, *p* < 0.001, indicating that the mean D-DIMER level decreased sharply from Time 1 to Time 2 and then slightly from Time 2 to Time 3.

There was also a significant main effect of group, F (1, 146) = 3604, *p* < 0.001, η2 = 0.961, indicating that the mean D-DIMER level differed significantly between the control group and the experimental group. The pairwise comparison revealed that the control group had a significantly higher mean D-DIMER level (M = 320.45, SE = 1.82) than the experimental group (M = 166.30, SE = 1.82).

**Figure 3:**
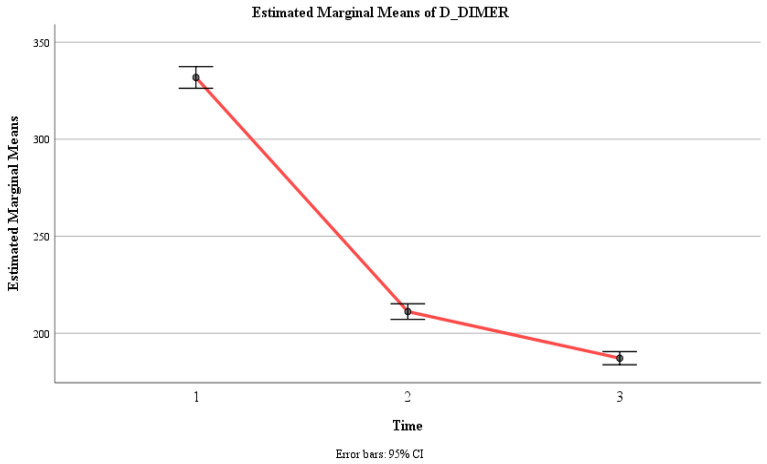
Estimated marginal means of D-DIMER levels over time for the control and experimental groups. Time effect mean values.

**Figure 4:**
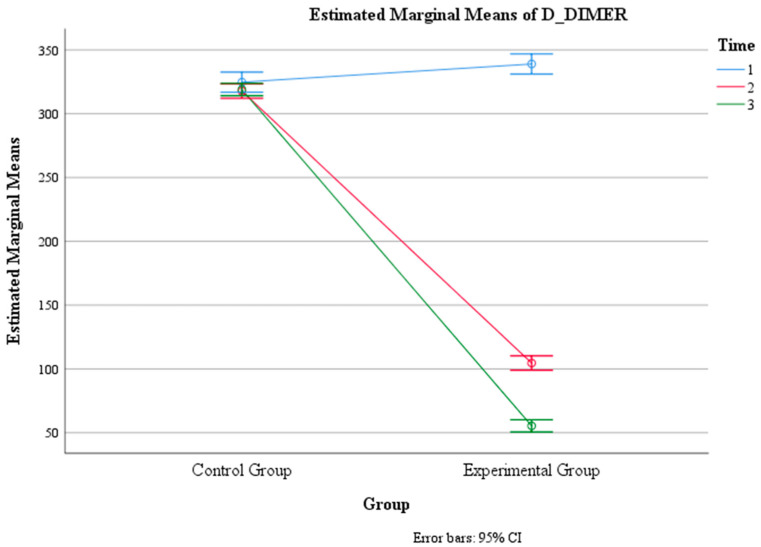
Estimated marginal means of D-DIMER levels over time for the control and experimental groups. Time group mean values.

Moreover, there was a significant interaction effect between time and group, F (1.86, 272.01) = 1088, *p* < 0.001, η2 = 0.882, indicating that the change in the mean D-DIMER level over time depended on the group. The within-subject contrast showed that this effect was quadratic, F (1, 146) = 295, *p* < 0.001, meaning that the difference in the mean D-DIMER level between the two groups varied across the three time points. Within the control group, there were no significant differences in D-DIMER levels between any of the three time intervals (F (2, 145) = 1.10, *p* = 0.335). Within the experimental group, there was a significant difference in D-DIMER levels between Time 1 (M = 338.96, SE = 3.991) and Time 2 (M = 104.60, SE = 2.86), between Time 1 and Time 3 (M = 55.35, SE = 2.43), and between Time 2 and Time 3 (F (2, 145) = 1832, *p* < 0.001) (Table 2, Figure 3 and Figure 4).

### 3.3. IL-6 Levels

There was a significant main effect of time, F (2, 292) = 75.76, *p* < 0.001, η2 = 0.342, indicating that the mean IL-6 level changed significantly across the three time points. The within-subject contrast showed that this effect was quadratic, F (1, 146) = 22.28, *p* < 0.001, meaning that the mean IL-6 level decreased from Time 1 to Time 2 and then slightly increased from Time 2 to Time 3.

There was also a significant main effect of group, F (1, 146) = 570.04, *p* < 0.001, η2 = 0.796, indicating that the mean IL-6 level differed significantly between the control group and the experimental group. The pairwise comparison revealed that the control group had a significantly higher mean IL-6 level (M = 19.70, SE = 0.38) than the experimental group (M = 6.83, SE = 0.38).

**Figure 5:**
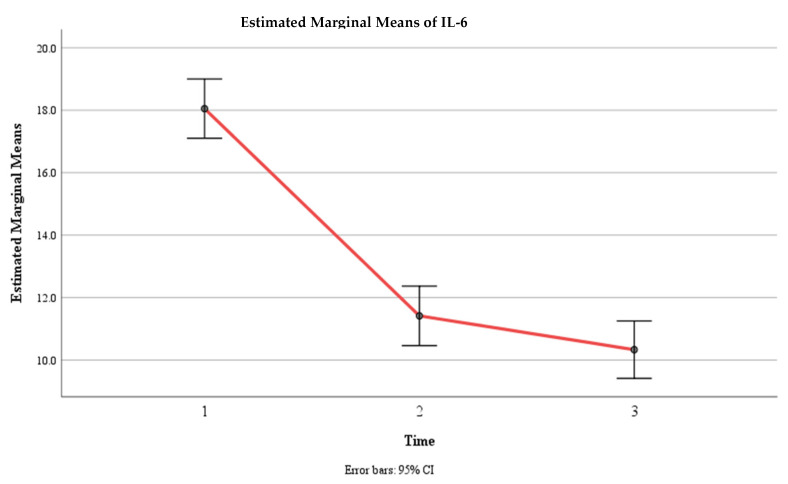
Estimated marginal means of IL-6 levels over time for the control and experimental groups. Time effect mean values.

**Figure 6:**
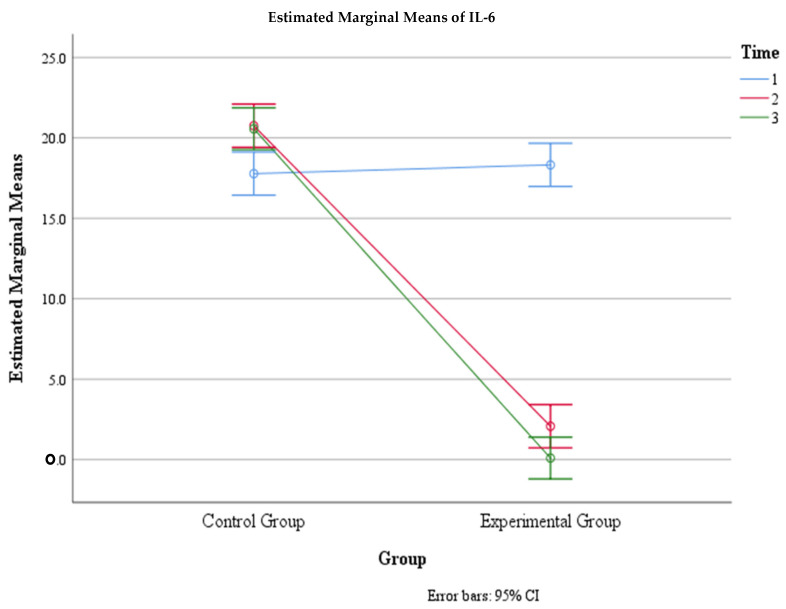
Estimated marginal means of IL-6 levels over time for the control and experimental groups. Time group mean values.

Moreover, there was a significant interaction effect between time and group, F (2, 292) = 147.15, *p* < 0.001, η2 = 0.502, indicating that the change in the mean IL-6 level over time depended on the group. The within-subject contrast showed that this effect was quadratic, F (1, 146) = 54.88, *p* < 0.001, meaning that the difference in the mean IL-6 level between the two groups varied across the three time points. Within the control group, there was a significant difference in IL-6 levels between Time 1 (M = 17.78, SE = 0.68) and Time 2 (M = 20.75, SE =0.68) (*p* < 0.01) and between Time 1 and Time 3 (M = 20.57, SE = 0.69) (*p* < 0.05), but there was no significant difference between Time 2 and Time 3 (*p* > 0.05). Within the experimental group, there was a significant difference in IL-6 levels between Time 1 (M = 18.32, SE = 0.68) and Time 2 (M = 2.08, SE = 0.68) (*p* < 0.01) and between Time 1 and Time 3 (M = 0.096, SE = 0.69) (*p* < 0.05), but there was no significant difference between Time 2 and Time 3 (*p* > 0.05) (Table 3, Figure 5 and Figure 6).

### 3.4. Erythrocyte Levels (n/µL)

There was a significant main effect of time, F (1.6, 237) = 45.83, *p* < 0.001, η2 = 0.239, indicating that the mean erythrocyte count changed significantly across the three time points. The within-subject contrast showed that this effect was not quadratic, F (1, 146) = 3.89, *p* > 0.05, meaning that the mean erythrocyte count did not follow a U-shaped or inverted U-shaped pattern over time.

There was also a significant main effect of group, F (1, 146) = 744, *p* < 0.001, η2 = 0.836, indicating that the mean erythrocyte count differed significantly between the control group and the experimental group. The pairwise comparison revealed that the control group had a significantly higher mean erythrocyte count (M = 16.13, SE = 0.22) than the experimental group (M = 7.83, SE = 0.22).

**Figure 7:**
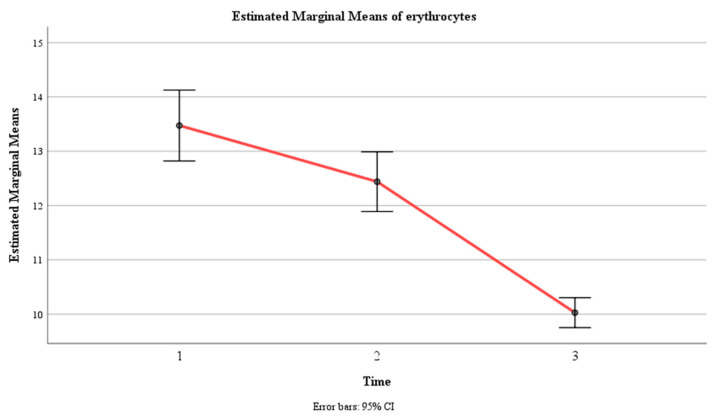
Estimated marginal means of erythrocyte levels over time for the control and experimental groups. Time effect mean values.

**Figure 8:**
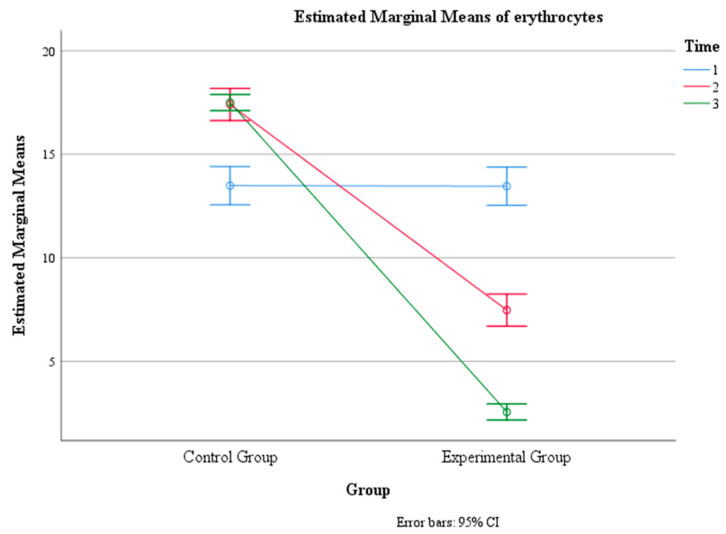
Estimated marginal means of erythrocyte levels over time for the control and experimental groups. Time group mean values.

Moreover, there was a significant interaction effect between time and group, F (1.6, 237) = 211, *p* < 0.001, η2 = 0.591, indicating that the change in mean erythrocyte count over time depended on the group. The within-subject contrast showed that this effect was quadratic, F (1, 146) = 12.24, *p* < 0.01, indicating that the difference in the mean erythrocyte count between the two groups varied across the three time points in a U-shaped or inverted U-shaped manner. The pairwise comparison showed that there was no significant difference in the mean erythrocyte count between the two groups at time 1 (*p* > 0.05), but there was a significant difference at Time 2 and Time 3 (*p* < 0.001), with the experimental group having a lower mean erythrocyte count than the control group at both time points (Table 4, Figure 7 and Figure 8).

### 3.5. Leukocyte Levels (n/µL)

There was a significant main effect of time, F (1.02, 149) = 146, *p* < 0.001, η2 = 0.500, indicating that the mean leukocyte count changed significantly across the three time points. The within-subject contrast showed that this effect was quadratic, F (1, 146) = 156.65, *p* < 0.001, meaning that the mean leukocyte count followed a U-shaped or inverted U-shaped pattern over time.

There was also a significant main effect of group, F (1, 146) = 167.24, *p* < 0.001, η2 = 0.534, indicating that the mean leukocyte count differed significantly between the control group and the experimental group. The pairwise comparison revealed that the control group had a significantly higher mean leukocyte count (M = 73.08, SE = 2.44) than the experimental group (M = 28.46, SE = 2.44).

**Figure 9:**
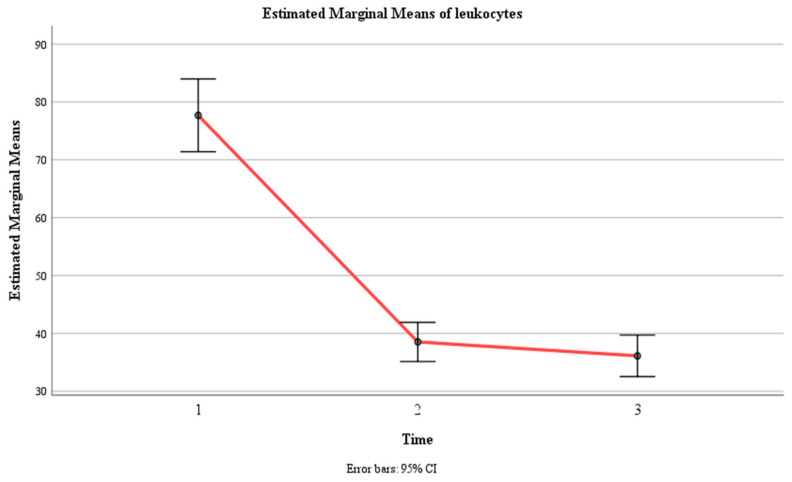
Estimated marginal means of leukocyte levels over time for the control and experimental groups. Time effect mean values.

**Figure 10:**
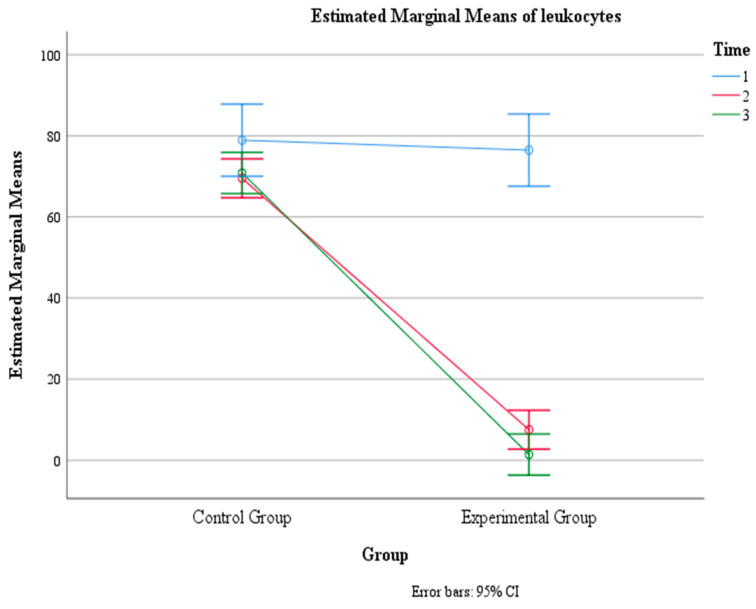
Estimated marginal means of leukocyte levels over time for the control and experimental groups. Time group mean values.

Moreover, there was a significant interaction effect between time and group, F (1.02, 149) = 90.43, *p* < 0.001, η2 = 0.382, indicating that the change in mean leukocyte count over time depended on the group. The within-subject contrast showed that this effect was quadratic, F (1, 146) = 58.41, *p* < 0.01, indicating that the difference in the mean leukocyte count between the two groups varied across the three time points in a U-shaped or inverted U-shaped manner. Within the control group, there was no significant difference between Time 1 (M = 78.91, SE = 4.50) and Time 2 (M = 69.51, SE = 2.43) (*p* > 0.05) and between Time 1 and Time 3 (M = 70.82, SE = 2.57) (*p* > 0.05), but there was a significant difference between Time 2 and Time 3 (*p* < 0.05). Within the experimental group, there was a significant difference between Time 1 (M = 76.47, SE = 4.50) and Time 2 (M = 7.51, SE = 2.43) (*p* < 0.001), between Time 1 and Time 3 (M = 1.39, SE = 2.57) (*p* < 0.001), and between Time 2 and Time 3 (*p* < 0.001) (Table 5, Figure 9 and Figure 10).

### 3.6. E. coli Levels (UFC/mL)

There was a significant main effect of time, F (1.5, 221) = 43.67, *p* < 0.001, η2 = 0.230, indicating that the mean *E. coli* count changed significantly across the three time points. The within-subject contrast showed that this effect was quadratic, F (1, 146) = 49.50, *p* < 0.001, indicating that the mean *E. coli* count followed a U-shaped or inverted U-shaped pattern over time.

There was also a significant main effect of group, F (1, 146) = 83.25, *p* < 0.001, η2 = 0.36, indicating that the mean *E. coli* count differed significantly between the control group and the experimental group. The pairwise comparison revealed that the control group had a significantly higher mean *E. coli* count (M = 420,045, SE = 15,621) than the experimental group (M = 218,477, SE = 15,621).

**Figure 11:**
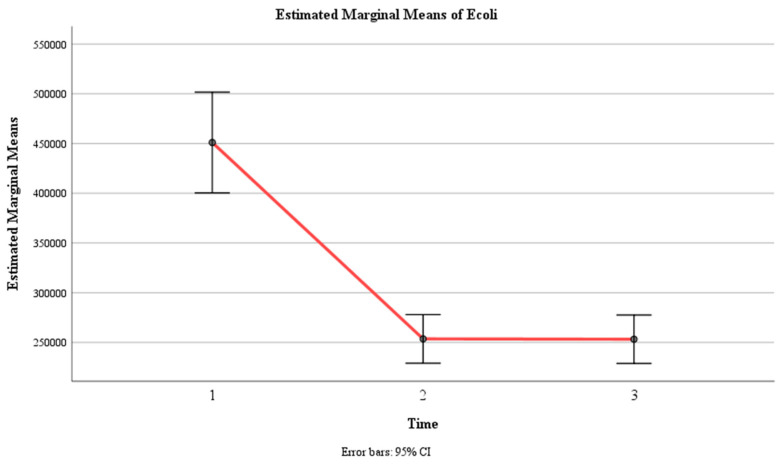
Estimated marginal means of *E. coli* levels over time for the control and experimental groups. Time effect mean values.

**Figure 12:**
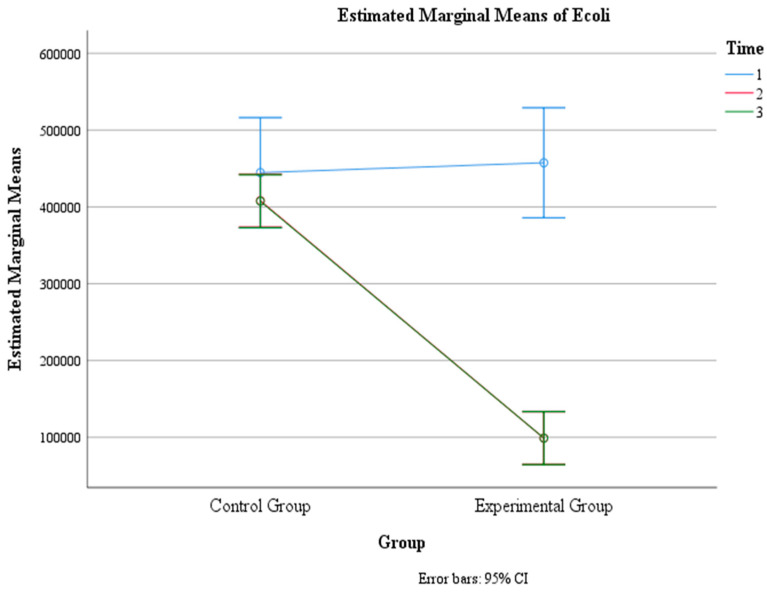
Estimated marginal means of *E. coli* levels over time for the control and experimental groups. Time group mean values.

Moreover, there was a significant interaction effect between time and group, F (1.5, 221) = 28.91, *p* < 0.001, η2 = 0.165, indicating that the change in the mean *E. coli* count over time depended on the group. The within-subject contrast showed that this effect was quadratic, F (1, 146) = 21.56, *p* < 0.001, meaning that the difference in the mean *E. coli* count between the two groups varied across the three time points in a U-shaped or inverted U-shaped manner. Within the control group, none of the pairwise comparisons were significant at all three time intervals (*p* > 0.05). Within the experimental group, there was a significant difference between Time 1 (M = 457,432, SE = 36,255) and Time 2 (M = 99,000, SE = 17,490) (*p* < 0.001) and between Time 1 and Time 3 (M = 99,000, SE = 17,446) (*p* < 0.001), but there was no significant difference between Time 2 and Time 3 (*p* > 0.05) (Table 6, Figure 11 and Figure 12).

In the statistical analysis, the elimination of *E. coli* was considered when the *E. coli* count was below the minimum number of 99,000 CFU/mL, which corresponds to *E. coli* < 100,000 CFU/mL. This threshold was chosen based on laboratory reports indicating that *E. coli* counts below 100,000 CFU/mL are reported as an “absence of *E. coli*”.

## 4. Discussion

The effect of EMFs on biological systems is well established. However, EMF-induced alterations were mostly deemed harmful [33]. Currently, researchers have shown that static or ELF electromagnetic fields have significant beneficial properties. The findings of the present study also resonate with these observations. We observed that treatment with ELF electromagnetic fields according to the osteopathic fundamentals of palpation and assessment can effectively manage cystitis and eliminate *E. coli* as early as 4 weeks.

From the obtained results, it was observed that initially, the control group had higher baseline average values of IL-6, D-DIMER, red blood cells, white blood cells, and *E. coli* compared to the experimental group, although both groups had values above the maximum threshold. However, in the control group, the values remained stable over time, while in the experimental group, they significantly decreased, reaching below the alert threshold for pathological manifestation. The initial steps of the sequence used may have a direct relationship with the white blood cells and their decrease after the BSBI treatment. This could be inferred from the available scientific literature, where there is evidence that different frequencies have specific effects on biological processes. For example, 1 Hz has been shown to influence vascular action in occluded vessels [26,34], 8 Hz affects nitric oxide and potassium chloride [35], 4 Hz impacts vagal action (including theta waves, norepinephrine, and acetylcholine), and 10 Hz may enhance hypoxia tolerance by altering the redox state of the extracellular matrix [36].

The present study showed that IL-6 significantly reduced in the treatment group compared to control over time. There is compelling evidence that ELFs and low-energy electromagnetic fields exert an anti-inflammatory effect through the upregulation of A2A and A3 adenosine receptors, leading to a reduction in the expression of inflammatory cytokines (TNFα, IL-1β, IL-8). EMF-induced upregulation of the A2A receptor occurs in various cell types and tissues, including neuronal cells, osteoblasts, and chondrocytes. Neutrophils exposed to EMFs (pulse length 1.3 ms, replication rate 75 Hz, 24 h, 0.2–3.5 mT) demonstrated a significant increase in A2A receptor signaling and the ability, upon treatment with adenosine agonists, to inhibit superoxide anion generation. Of particular interest for ischemic damage control are the inhibitory effects of EMF exposure on hypoxia-inducible factor 1α (HIF1α) expression in microglial cells [37]. Microglial cell activation during ischemia and reperfusion leads to the amplification of danger signals, resulting in strong inflammatory responses that contribute significantly to tissue damage.

It has been demonstrated that frequencies of 50 Hz, 10 Hz, 5 Hz, 55 Hz, and 38 Hz with rectangular waves and intensities up to 50 mT can significantly decrease serum levels of IL-9, IL-1, and TNF-α and increase IL-10 levels. However, it is known that by default, IL-6 decreases as an inhibitor of TNF-α [18]. There is evidence showing that an ELF with a frequency of 10 Hz and an intensity of 1 mT can markedly increase resistance to hypoxia [36].

The effects of a pulsed EMF (PEMF) on tendon cell response in an environment conditioned by IL-1β and IL-6 have been explored, highlighting the potential regulation of inflammation and tissue repair [19]. Furthermore, a PEMF can attenuate the progression of osteoarthritis in a murine model through the inhibition of TNF-α and IL-6 signals, which are crucial pro-inflammatory cytokines in the pathogenesis of osteoarthritis [20]. The PEMF also suppresses the transcription of IL-6 in bovine nucleus pulposus cells [38].

The effects of a static magnetic field on IL-6 secretion in human colon myofibroblasts have been examined, providing insight into the modulation of inflammation at the intestinal level [39]. Additionally, the effect of the PEMF on the expression of IL-6 in intervertebral disc cells through the Nuclear Factor-κB and Mitogen-Activated Protein Kinase p38 pathways further underscores the potential anti-inflammatory effects of the PEMF [40]. The effects of exposure to 60 Hz on the production of IL-1 and IL-6 have been observed, highlighting possible mechanisms through which electromagnetic fields can influence immune responses [41].

It is known that a low-frequency electromagnetic field can offer protection against ischemia/reperfusion injuries in the rat heart [42]. Adenosine A2A receptors have been found to be involved in the reparative response of tendon cells to the PEMF, suggesting a mechanism through which the PEMF can promote tissue repair [43], as well as effects on cytokine production in rats [44].

The present study also demonstrated a significant reduction in *E. coli* levels following treatment. Previously, studies have investigated the effect of electromagnetic radiation on *E. coli* in vitro. Azeemi et al. reported that the most profound inhibitory effects were observed with radiation in the visible range of 538 nm (green), which proved to be bactericidal, and radiation in the visible range of 590 nm (yellow), which was bacteriostatic [45]. Justo et al. studied the influence of an ELF on *E. coli* cultures during submerged fermentation. *E. coli* cultures exposed to 0.1 T for 6.5 h showed changes in their vitality compared to unexposed cells, which were 100 times higher than the control [46]. Another study showed that exposure of *E. coli* to 0.3 Hz for 90 min was the most inhibitory frequency, where bacterial growth was inhibited by 42.3% [31]. In a study by Bayır et al., the effect of the ELF-EMF on the growth of Staphylococcus aureus and *Escherichia coli* O157:H7 was studied. Samples exposed to the ELF-EMF showed a statistically significant decrease (*p* < 0.05) in colony-forming ability compared to controls, especially with longer exposure times. Exposure to 4 mT-20 Hz ELF for 6 h produced the maximum inhibition of CFUs compared to controls for both microorganisms (95.2% for *S. aureus* and 85% for *E. coli*) [32].

Furthermore, there is evidence of the action of 15 Hz on cell communication (activity on Ca-ATPase) [23] and of 50 Hz and 60 Hz on inflammation, oxidative stress, cytoprotection, and bacterial growth [20,38,39,40]. Therefore, the action on white blood cells at 6Hz at different intensities and waveforms remains to be investigated. It is conceivable that submultiples of 60 Hz, such as 6 Hz and 3 Hz, may be effective on lymphocyte action [47], and since the growth of *E. coli* increases above 50 Hz [48], it was decided to use a submultiple instead of the frequency already examined previously, except in the last step of BSBI, which offers cytoprotection to the involved tissues.

## 5. Conclusions

Based on the obtained results, it can be inferred that in chronic drug-resistant urinary tract infections, attention should be focused not only on the pathogen but on the entire infectious process, correlating antioxidant activity, inflammation, tissue repair capacity, and antibacterial effects. Treatment with sequentially sequenced electromagnetic fields (BSBI) applied on osteopathic palpation landmarks, modulated at extremely low frequencies and intensities, not only appears to offer an effective alternative for the symptoms of chronic bacterial cystitis caused by drug-resistant *E. coli* but also demonstrates a potent antibacterial, antioxidant, anti-inflammatory, and immunomodulatory effect without causing side effects. Regarding the symptoms of chronic cystitis, it was observed that in the experimental group, they were no longer present at the end of the treatment or during the follow-up period, whereas they were present in the control group. Further studies are necessary to confirm these results and to delve into the mechanisms underlying the effectiveness of the treatment.

## Figures and Tables

**Table 1:** Descriptive statistics for the variables (N = 148).

Variables	M	SD	Min	Max
BMI	21.26	1.66	18.5	24.2
pH	6.64	0.70	5.5	7.9
Specific Gravity	1015.58	5.63	1006	1025
Urobilinogen mg/dL	0.81	0.45	0	1.5

**Table 2:** Means (standard errors) for D-DIMER levels (ng/mL) as a function of time and group.

Group	Time 1	Time 2	Time 3	F (df)	*p*-Value
Experimental	338.96 (3.99)	104.60 (2.86)	55.35 (2.43)	1088 (1.86, 272.01)	<0.001
Control	324.72 (3.99)	317.72 (2.86)	318.92 (2.43)		
Time Only	331.84 (2.82)	211.16 (2.02)	187.14 (1.72)	1195 (1.86, 272.01)	<0.001

**Table 3:** Means (standard errors) for IL-6 levels (pg/mL) as a function of time and group.

Group	Time 1	Time 2	Time 3	F (df)	*p*-Value
Experimental	18.32 (0.68)	2.08 (0.68)	0.096 (0.69)	147.15 (2, 292)	<0.001
Control	17.78 (0.68)	20.75 (0.68)	20.57 (0.69)		
Time Only	18.05 (0.48)	11.42 (0.48)	10.33 (9.41)	75.76 (2, 292)	<0.001

**Table 4:** Means (standard errors) for erythrocyte levels (n/µL) as a function of time and group.

Group	Time 1	Time 2	Time 3	F (df)	*p*-Value
Experimental	13.46 (0.47)	7.47 (0.39)	2.56 (0.20)	211 (1.6, 237)	<0.001
Control	13.49 (0.47)	17.41 (0.39)	17.50 (0.20)		
Time Only	13.47 (0.33)	12.44 (0.28)	10.03 (0.14)	45.83 (1.6, 237)	<0.001

**Table 5:** Means (standard errors) for leukocyte levels (n/µL) as a function of time and group.

Group	Time 1	Time 2	Time 3	F (df)	*p*-Value
Experimental	76.47 (4.50)	7.51 (2.43)	1.39 (2.57)	90.43 (1.02, 149)	<0.001
Control	78.91 (4.50)	69.51 (2.43)	70.82 (2.57)		
Time Only	77.69 (3.19)	38.51 (1.72)	36.11 (1.82)	146.13 (1.02, 149)	<0.001

**Table 6:** Means (standard errors) for *E. coli* levels (UFC/mL) as a function of time and group.

Group	Time 1	Time 2	Time 3	F (df)	*p*-Value
Experimental	457,432 (36,255)	99,000 (17,490)	99,000 (17,446)	28.91 (1.5,221)	<0.001
Control	444,595 (36,255)	408,108 (17,491)	407,432 (17,446)		
Time Only	451,013 (25,636)	253,554 (12,368)	253,216 (12,336)	43.67 (1.5,221)	<0.001

## Data Availability

The data presented in this study are available on request from the corresponding author.
